# Effects of *Stephania hainanensis* alkaloids on MSU-induced acute gouty arthritis in mice

**DOI:** 10.1186/s12906-021-03364-5

**Published:** 2021-07-20

**Authors:** Hao-fei Fan, Xing-yue Fang, Hao-lin Wu, Yi-qian Xu, Li-chong Gong, Dao-rui Yu, Hao Jia, Xiao-liang Tang, Qi-bing Liu

**Affiliations:** 1 Department of Pharmacology, School of Basic and Life Science, Hainan Medical University, Haikou, 571199 China; 2 HMC Cancer Institute, The First Affiliated Hospital of Hainan Medical University, Haikou, China; 3 Martinos Center for Biomedical Imaging, Massachusetts General Hospital and Harvard Medical School, Boston, MA 02129 USA; 4 School of Traditional Chinese Medicine, Hainan Medical University, Haikou, 571199 China

**Keywords:** Gouty arthritis, Monosodium urate crystal, *Stephania hainanensis*, Inflammasome

## Abstract

**Background:**

Gout is initiated by the precipitation of monosodium urate (MSU) crystals within the joints and soft tissues, and it can eventually cause acute or chronic arthritis. MSU crystals trigger, amplify, and maintain a strong inflammatory response through promoting proinflammatory activity. In this study, the therapeutic effects of *Stephania hainanensis* (*S. hainanensis*) total alkaloid (SHA) were tested and evaluated on MSU-induced acute gouty arthritis in a mouse model.

**Methods:**

After oral administration of SHA (10 or 20 mg/kg) or the antigout medicine colchicine (0.5 mg/kg) once daily for 3 consecutive days, MSU crystals suspended in saline (2.5 mg/50 μl) were intradermally injected into the right paw of the mice. Then, SHA and colchicine were administered for another 2 days. During this period, swelling of the ankle and clinical scores were measured at 12, 24, and 48 h postinjection. After the mice were euthanized, inflammatory cytokine expression and paw tissue inflammation-related gene and protein expression, and a histopathological analysis was performed.

**Results:**

SHA had obvious therapeutic effects on MSU-induced acute gouty arthritis in mice. SHA alleviated ankle swelling and inhibited the production of cytokines, such as IL-1β and TNF-α. In addition, NLRP3, Caspase-1 and IL-1β, which are activated by MSU were also suppressed by SHA. The histological evaluation showed that SHA relieved the infiltration of inflammation around the ankle.

**Conclusions:**

These results suggest that SHA is capable of anti-inflammatory activities and may be useful for treating gouty arthritis.

**Supplementary Information:**

The online version contains supplementary material available at 10.1186/s12906-021-03364-5.

## Background

Gout is an inherited disorder of purine metabolism that causes hyperuricemia and inflammatory arthritis, and it is very common worldwide [1]. Gouty patients usually suffer from unbearable pain and joint swelling, which seriously affect the normal life and might even cause disability [2]. It is estimated that the prevalence ranges from < 1 to 6.8% and that the incidence is 0.58–2.89 per 1000 person-years [3], especially among the middle-aged and elderly population; additionally, the prevalence and incidence are increasing. The main cause of its acute phase is that monohydrate sodium urate (MSU) crystals are formed and deposited in the joint and periarticular tissues [4]. MSU crystals can be seen on biopsy under synovial microscopy, and a tophus can form in the joints with repeated episodes of the disease [5].

The main goals of gouty arthritis therapy are to alleviate pain, reduce inflammatory responses to MSU crystals, and ameliorate symptoms rapidly and safely [6]. Normally prescribed gouty arthritis medications include nonsteroidal anti-inflammatory drugs (NSAIDs), analgesic drugs, and colchicine [7]. Colchicine is regularly used in the treatment of gout attacks, has specific clinical efficacy and inhibits neutrophil recruitment and infiltration [8]. Unfortunately, these drugs might cause unwanted side effects, such as gastrointestinal bleeding, diarrhoea and vomiting [9]. Natural alternative anti-inflammatory supplements have been used to mediate the inflammatory process and often induce fewer side effects [10]. Therefore, we focused our research on the discovery of a drug with anti-inflammatory activity from natural resources.

*Stephania hainanensis* H. S. Lo et Y. Tsoong is an annual herb distributed in Baisha, Qiongzhong, Danzhou and other places in Hainan Province, China [11, 12]. The tuberous roots of *Stephania hainanensis* (*S. hainanensis*) are used as a Chinese folk herb medicine for the treatment of inflammation, trauma, pain and fever [13]. However, the effects of the internal active ingredients of *S. hainanensis* as a medicinal plant are still uncertain. *Stephania hainanensis* alkaloids (SHAs) have many pharmacological activities, such as sedative and anti-addictive actions and anti-inflammatory effects [14]. The analgesic and anti-inflammatory effects of SHA have also been confirmed in experiments with animal models, such as the hot-plate test, acetic acid-induced twisting reaction, and dimethylbenzene-induced ear swelling in mice [15]. To date, the therapeutic effects of SHA on acute gouty arthritis have not been reported. Considering that SHA possesses anti-inflammatory and analgesic activities in both human and animal inflammatory models, we hypothesized that it would be effective in treating inflammation and pain in gout arthritis. In this study, we examined the therapeutic effects of SHA in the treatment of inflammation and pain responses in MSU-induced gouty arthritis in a mouse model and evaluated the involved mechanisms involved in its therapeutic effects.

## Methods

### SHA total alkaloid preparation and identification

The roots of *Stephania hainanensis* were collected from Wuzhi Mountain in Hainan Province and identified by Prof. Niankai Zeng. A voucher specimen was deposited in the Herbarium of Hainan Medical University (No. SH20191008). Fresh roots (10 kg) were cut into pieces and dried in an oven at less than 50 °C. The dried plant (1.0 kg) was extracted by distilled water containing 1.0% H_2_SO_4_ (5 L) through decoction twice (1 hour each time), and then the pH value of the acidic water extract was adjusted to 10 by adding ammonia. The basic extract was partitioned by chloroform three times and concentrated under reduced pressure to yield the extract (100.0 g). Next, the extract was identified by TLC under UV 254 nm to confirm the total alkaloid obtained from *S. hainanensis*.

### LC-QTOF-MS/MS analysis

The UPLC-QTOF/MS analysis was performed on an Agilent 1290 Infinity II UHPLC system coupled to an Abscix Triple TOF/MS instrument. Chromatographic separation was achieved using a Phenomenex Kinetex XB-C18 (2.1 mm × 50 mm, 1.7 μm particle) analytical column operated at 40 °C. The mobile phase consisted of water (A) and methanol (B) at a flow of 0.35 ml·min^− 1^. The gradient started from 90% A in 0.5 min, followed by 90% A to 10% A in 11.5 min, and held 10% A for 2 min, then returned the initial gradient composition and allowed to equilibrate for 2 min. Mass spectrometric analyses were collected in positive mode with full scan mode from 100 to 1000 m/z. The spray voltage was 5.5 kV, and the desolvation temperature was 550 °C. The pressures of the inner coaxial nebulizer N2 gas (GS1), dry N2 gas (GS2), and curtain N2 gas (CUR) were 50, 50, and 30 psi, respectively.

### Animal model of acute gouty arthritis with MSU crystals in mice

MSU crystals (CAS number: 1198-77-2) were purchased from InvivoGen (San Diego, CA) and resuspended in 2.5% Tween 80-sterile saline to 50 mg/ml as the stock solution. Kunming (KM) male mice (8 weeks old, 22–24 g body weight) were purchased from Changsha Tianqin Biotechnology Co., Ltd. (Hunan, China, certificate number: SCXK (Xiang) 2014–0012). The mice were controlled in cages (at a density of 5 mice per cage) under an ambient temperature of 23 ± 2 °C, relative humidity of 55 ± 10% and a 12 h:12 h light-dark cycle with free access to water and food. After acclimation for 1 week, 50 KM male mice were randomly divided into five groups (*n* = 10 mice per group), and random numbers were generated using the *RAND()* function of Microsoft Excel. The groups included the control group, MSU injection group, oral colchicine (Col) group (0.5 mg/kg) [16], and oral SHA treated group (10 and 20 mg/kg, respectively). SHA and colchicine were administered once daily for 3 days before the MSU crystal suspension (2.5 mg/50 μl) was intraarticularly injected into the right paw of the mice while the left paw was injected with saline as a control [17]. All injections were performed with mice that were anaesthetised with isoflurane. After the injection, the drugs were administered once daily for another 2 days. Finally, the mice were anaesthetized by intraperitoneal injection of pentobarbital sodium (50 mg/kg), blood was collected from the retroorbital venous plexus, and the mice were sacrificed via exposure to CO_2_. The right paw tissue was separated from the skin and bone, washed with PBS and stored at − 70 °C for further analysis. All experimental operations with mice followed the National Guidelines for Experimental Animal Care and Use in China. The associated animal protocols were approved by the Animal Ethics Committee of Hainan Medical University (Haikou, China).

### Ankle swelling measurement and evaluation

Ankle swelling was measured at different time points using traceable digital calipers (Fisher Scientific). Based on the degree and extent of tissue swelling near the ankle, we independently scored the degree of arthritis in mice (normal was scored as “0”, slight swelling and/or erythema was scored as “1”, moderate swelling/erythema was scored as “2”, severe oedema/erythema was scored as “3”, and excessive oedema spreading all over the paw was scored as “4”).

### Tissue inflammatory cytokines and blood leukocyte determination

The paw tissue levels of cytokines (IL-1β and TNF-α) were measured using ELISA kits from Beyotime Biotechnology (Shanghai, China). Measurements were performed according to the manufacturer’s protocols. Blood leukocyte classification and counting were determined by a Beckman DxH 900 haematology analyser.

### Real-time PCR detection

Total RNA from mouse paw tissue was isolated by TRIzol (Beyotime, Shanghai, China). After the purity test was qualified, 1 μg of total RNA was reverse transcribed to generate a cDNA library. Real-time quantitative PCR was implemented with different primers and a SYBR® Green RT-PCR reagent kit (Millipore, MA, USA). All the primers were synthesized by Sangon Biotech (Shanghai, China) Co., Ltd., and the sequences are listed in Table 1. The mRNA expression of NLRP3, Caspase-1, IL-1β and TNF-α was normalized to the ratio of GAPDH. All real-time PCR experiments were repeated three times.

**Table 1:** Real-time PCR primer sequence

Gene	Forward primer (5′ to 3′)	Reverse primer (5′ to 3′)
NLRP3	GGGACTCAAGCTCCTCTGTG	GAGGCTCTGGTTATGGGTCA
Caspase-1	TCCGCGGTTGAATCCTTTTCAGA	ACCACAATTGCTGTGTGTGCGCA
IL-1훽	CCTCGTGCTGTCGGACCCATA	CAGGCTTGTGCTCTGCTTGTGA
TNF-훼	CAAAGGGAGAGTGGTCAGGT	GGCAACAAGGTAGAGAGGC
GAPDH	TGTCATACTTGGCAGGTTTCT	CGTGTTCCTACCCCCAATGT

### Western blot analysis

The hind paw tissues were collected from euthanized mice and shredded with surgical scissors in disposable culture dishes placed on dry ice. All tissue pieces (1 mm × 1 mm) were placed in 1.5 ml tube, added to1 ml RIPA lysis buffer (Santa Cruz) and then homogenized with a bead microtube homogenizer (Sigma-Aldrich) for 45 s at a speed of 5 m/s. Tubes were then kept on ice for 30 min and centrifuged at 12,000 g for 10 min at 4 °C. Afterwards, the supernatants were gathered in individual tubes and stored at − 80 °C. For Western blot detection, total paw-extracted proteins were quantified by a Pierce™ BCA Protein Assay Kit, and 80 μg of protein was loaded on a 5% concentrated gel and 10–12% segregated polyacrylamide gels. After 1 h of electrophoresis, the separated proteins were transferred to PVDF membranes (100 V, 60 min) and blocked with 5% skimmed milk for 1 h. Membranes were then incubated with anti-NLRP3 (D4D8T) (Cell Signaling Technology), anti-caspase-1 p10 (M-20) (Santa Cruz), anti-IL-1β (3A6) (Cell Signaling Technology) and anti-GAPDH antibodies (Abcam) at 4 °C overnight. The Goat anti-rabbit IgG H&L (HRP) was incubated with secondary antibodies at a concentration of 1:1000 (RT, 1 h) before reaction with Pierce™ ECL Plus Western Blotting Substrate. The greyscale values of the bands were quantified by Image-Pro Plus Version 7.0 software (Media Cybernetics).

### Histological studies

Mouse ankle joints were fixed in 4% paraformaldehyde, decalcified for 14 days at room temperature in 0.5 M EDTA-PBS solution (pH 7.8) and washed with PBS for 3 times. After embedding in paraffin, a Leica RM2235 Manual Rotary Microtome was used to prepare the 5-μm sections, which were stained with a haematoxylin-eosin dyeing system. Images were acquired using an Olympus BX60 Upright Compound Microscope at 10X and 40X magnifications.

### Statistical analysis

All data were expressed as the mean ± SD. Group differences were evaluated by one-way analysis of variance followed by Dunnett’s multiple comparison tests using GraphPad Prism version 8.0 software (GraphPad Software Inc., La Jolla, CA). Statistical significance was accepted at p<0.05.

## Results

### Preparation and identification of SHA total alkaloid

**Fig. 1:**
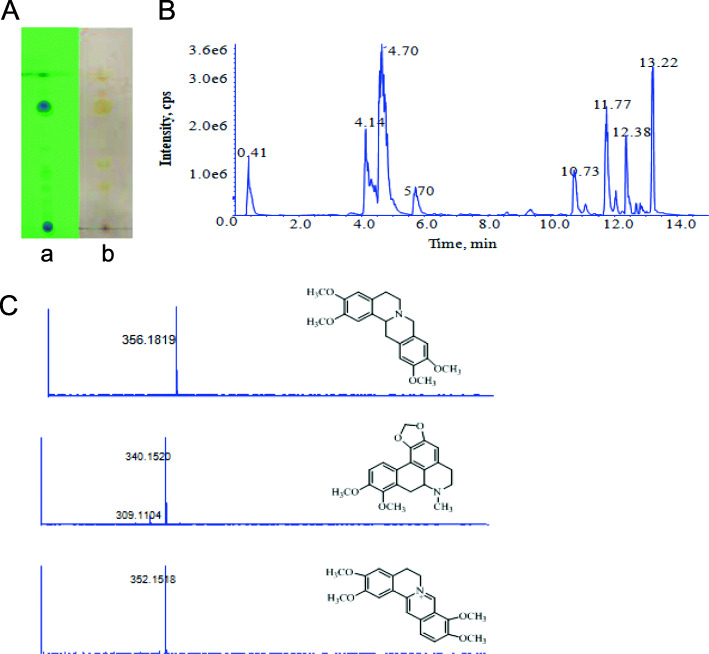
TLC and LC-MS profiles of *S. hainanensis* total alkaloids*.* **A** TLC was monitored under UV 254 nm (**A**) and sprayed with Dragendroff’s reagent (**B**). **B** The LC-MS profile of total alkaloids obtained from *S. hainanensis*. **C** The MS data and structures of identified alkaloids in total alkaloids of *S. hainanensis*

The TLC profile of the total alkaloid of *S. hainanensis* is shown in Fig. 1a. The spots in the TLC were monitored under UV 254 nm (Fig. 1a) and sprayed by Dragendroff’s reagent of potassium bismuth iodide (Fig. 1a-b). From the results of spraying with Dragendroff’s reagent, many alkaloids were confirmed by checking the positive reactions between alkaloids and Dragendroff’s reagent. The LC-MS profile of the total alkaloid of *S. hainanensis* is shown in Fig. 1b. The MS data and structures of identified alkaloids of SHA are shown in Fig. 1c. The identified alkaloids obtained from *S. hainanensis* extracts are listed in Table 2.

**Table 2:** The identified alkaloids obtained from *S. hainanensis* extracts

No	Retention time (min)	[M + H]^+^	Compounds
1	0.41	380.0947	Unidentified
2	4.1	356.1819	Xylopinine
3	4.7	340.1520	Crebanine
4	5.7	352.1518	Plamatine
5	10.73	396.8802	Unidentified
6	12.38	270.0885	Unidentified

### Effect of SHA on MSU crystal-induced paw oedema

**Fig. 2:**
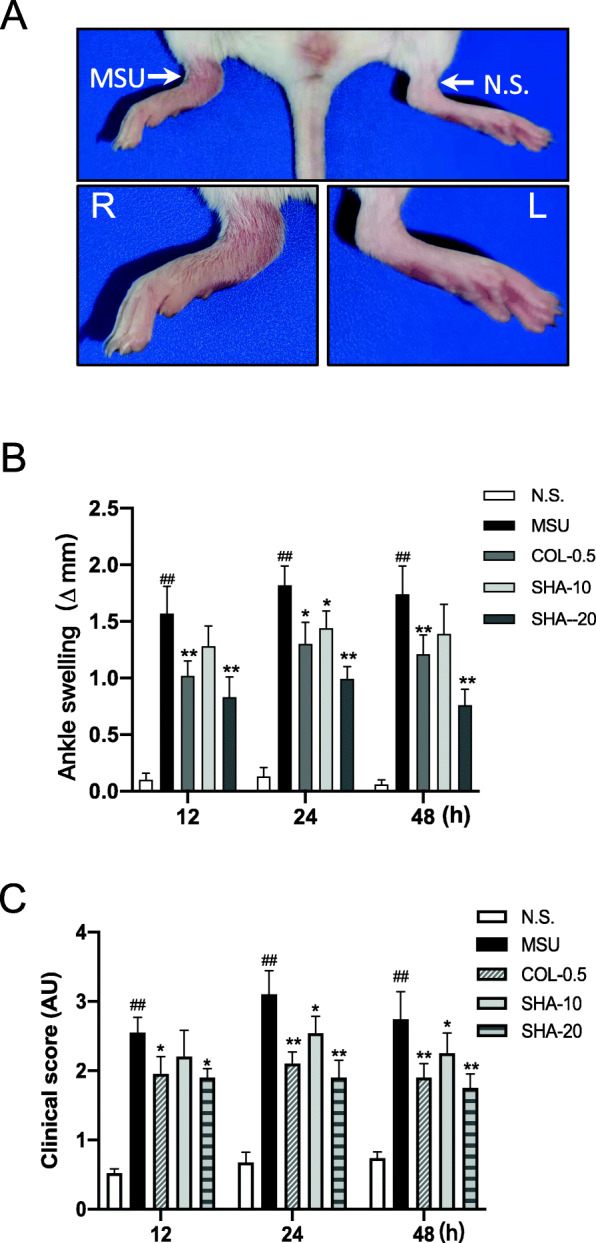
Effect of SHA on ankle swelling and clinical score of MSU-induced acute gouty arthritis in mice. **A** Representative photos of MSU-induced mouse ankle joint swelling captured at 24 h postinjection. Images at the lower panel are magnified pictures of the region indicated by the white arrows in the upper image. **B** & **C**, N.S., control saline group; MSU, MSU crystal-injected mice; COL-0.5, mice treated with MSU and 0.5 mg/kg colchicine; SHA-10, mice treated with MSU and 10 mg/kg SHA; SHA-20, mice treated with MSU and 20 mg/kg SHA. **B** MSU crystal-induced mouse ankle swelling at different times. **C** The clinical score evaluation for all mice after the ankle swelling measurement. All values are presented as the mean ± SD (*n* = 10). ^##^ *P* < 0.01 versus the saline group; * *P* < 0.05, ** *P* < 0.01 versus the MSU group

To evaluate the ankle swelling, the thickness of the claw palms of the control and treated mice was measured. As shown in Fig. 2a, after the injection of MSU crystals for 24 h, significant redness, oedema, and deformity were observed at the right ankle joints of the mice compared with those of the mice given the saline injection. The ankle swelling and clinical score of MSU crystal injection were increased significantly and found to be alleviated after the treatment with SHA (20 mg/kg) and colchicine (0.5 mg/kg), as shown in Fig. 2b and c. These results suggest that MSU crystal-induced ankle swelling was suppressed by SHA.

### Effects of SHA on proinflammatory cytokines and blood leukocyte production

**Fig. 3:**
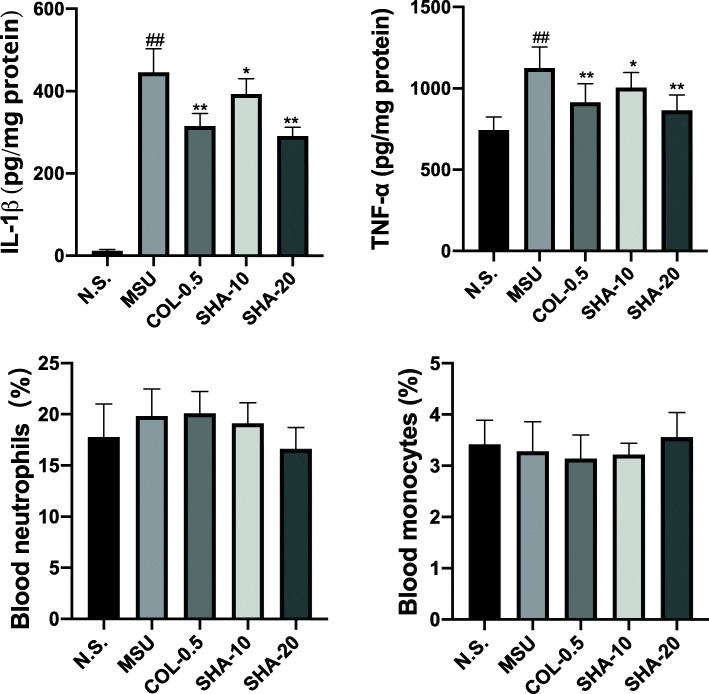
Effects of SHA on inflammatory cytokines in gouty ankle tissue and inflammatory cells in peripheral blood. Mice groups information are same as before. **A** & **B** Determination of IL-1β and TNF-α by ELISA. **C** & **D** Blood neutrophil and monocyte percentages measured by biochemical analysis. All values are presented as the mean ± SD (*n* = 10). ^##^ *P* < 0.01 versus the saline group; * *P* < 0.05, ** *P* < 0.01 versus the MSU group

We determined the paw tissue levels of the cytokines IL-1β and TNF-α to investigate the anti-inflammatory effect of SHA. The levels of these two proinflammatory cytokines were detected by ELISA after the MSU crystals were injected intradermally for 48 h. Simultaneously, blood leukocyte classification and numbers were determined. The ELISA results (Fig. 3a and b) showed that MSU induced a significant elevation in IL-1β and TNF-α levels; however, treatment with SHA significantly downregulated the production. Colchicine also significantly decreased the proinflammatory cytokine levels compared with those in the MSU group. In addition, there was no significant change in the blood ratio of neutrophils and monocytes for all groups. The results clearly showed that SHA treatment suppressed the major inflammatory cytokines (TNF-α and IL-1β), which are essential in the initiation and progression of gouty arthritis.

### SHA inhibited NLRP3 inflammasome activation and IL-1β in mice treated with MSU crystals

**Fig. 4:**
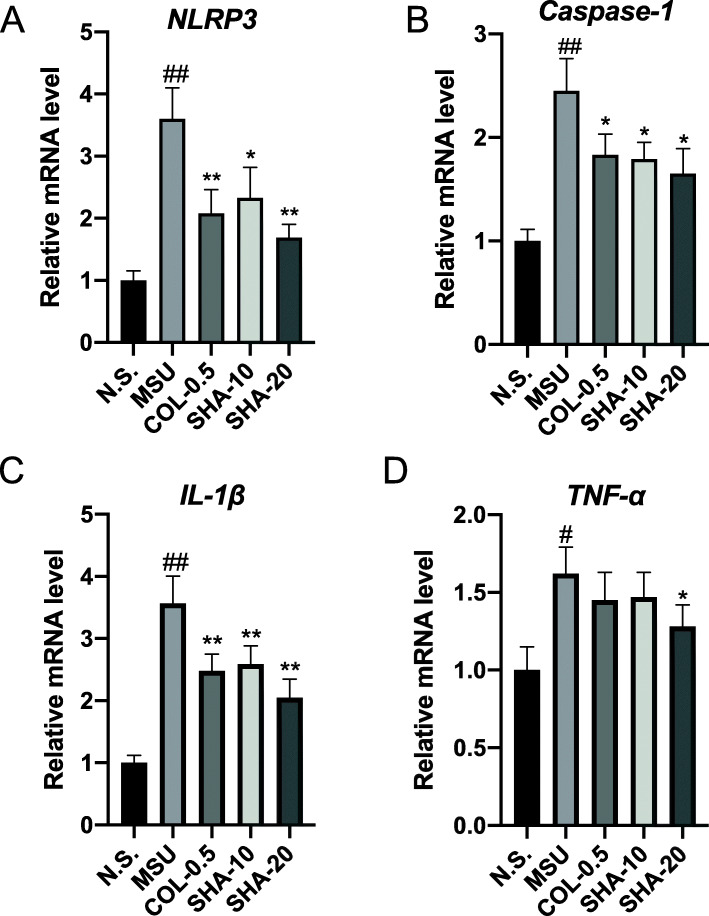
Effects of SHA on the mRNA expression of *NLRP3, Caspase-1, IL-1β* and *TNF-α* in gouty ankle tissue. **A** NLRP3, **B** Caspase-1, **C** IL-1β and **D** TNF-αmRNAs were measured by real-time PCR, and the expression levels were normalized to GAPDH. All data are presented as the mean ± SD (*n* = 5). ^#^ *P* < 0.05, ^##^ *P* < 0.01 versus the saline group; * *P* < 0.05, ** *P* < 0.01 versus the MSU group

**Fig. 5:**
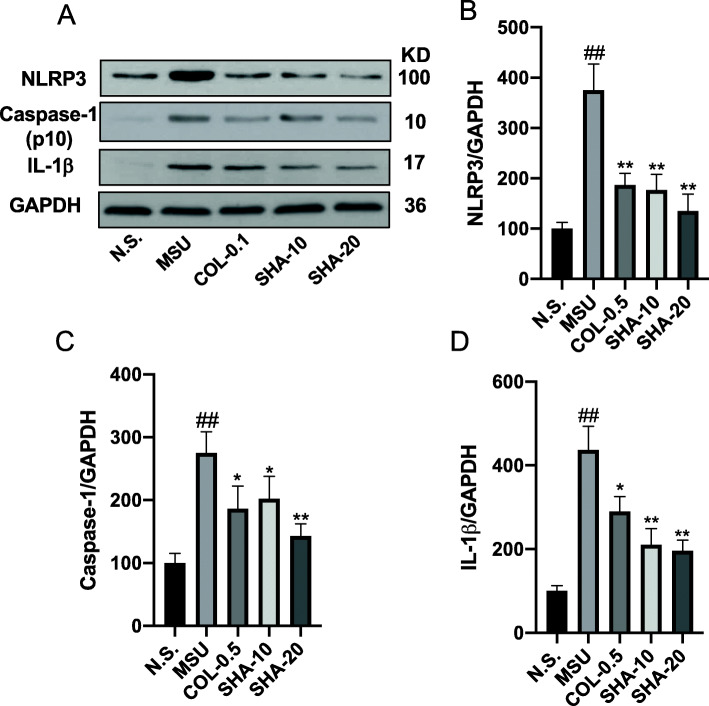
NLRP3, Caspase-1, and IL-1β expression in gouty ankle tissue of MSU- induced mice. Protein expression of NLRP3 and substrates in mouse ankle tissue was analysed by Western blotting after MSU crystals suspension (2.5 mg/50 ml) was injected intradermally for 48 h (**A**). **B-D** the greyscale values of the bands were quantified by Image-Pro Plus Version 7.0 and GAPDH was used as a loading control. The data shown are the mean ± SD of at least three independent experiments. ^##^ *P* < 0.01 versus the saline group; * *P* < 0.05, ** *P* < 0.01 versus the MSU group

To investigate the mechanisms underlying the anti-inflammatory effects of SHA, the mRNA expression levels of inflammatory cytokines (TNF-α and IL-1β) and inflammasome components (NLRP3 and caspase-1) were measured in MSU-induced paw tissue via real-time PCR analysis. As shown in Fig. 4, a marked increase in the mRNA expression levels of inflammatory cytokines (TNF-α and IL-1β), NLRP3, and caspase-1 was observed in the paw tissue of MSU-induced mice compared with the levels observed in the control group. Conversely, compared with the MSU group, the MSU group treated with SHA showed a dose-dependent decrease in the transcriptional levels of inflammatory cytokines (TNF-α and IL-1β), NLRP3, and caspase-1. These findings indicate that the decreased mRNA levels of NLRP3 and caspase-1 are responsible for the reduction in cytokine production. Next, we examined the effects of SHA on NLRP3 inflammasome activation by Western blotting. Consistent with our real-time PCR results, the Western blot analysis indicated that SHA and colchicine both significantly attenuated the overexpression of NLRP3, Caspase-1, and IL-1β protein in ankle joint tissues induced by MSU crystals (Fig. 5). Taken together, the results above indicate that SHA inhibits NLRP3 inflammasome activation induced by MSU in ankle joint tissues in mice.

### SHA improved ankle joint lesions in mice treated with MSU crystals

**Fig. 6:**
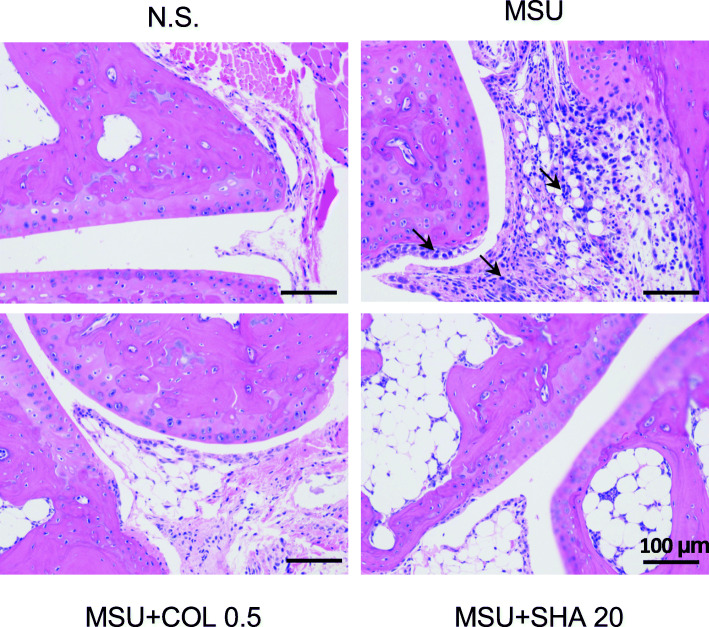
Effect of SHA on MSU crystal-induced ankle gouty arthritis in mice. The Haematoxylin-eosin-stained sections of ankle joints from the different mouse groups were obtained at 48 h after MSU injection. The arrows indicate the thickening of synovial and infiltrating inflammatory cells, bars = 100 μm

A histopathological analysis was performed by evaluating the lesions of ankle joints (periosteum and cartilage) in mice treated with MSU crystals to assess whether SHA could improve the histological lesions in mice. As shown in Fig. 6, in the control group, the cartilage and periosteum of the ankle joints in mice showed no degeneration and the structure was normal. Obvious pathological changes were observed in the ankle joint of the mice in the model control group, including evident synovial thickening, chondrocyte vacuole deterioration, inflammatory cell accumulation, swelling of interstitial tissue, and inflammatory exudation. All SHA and colchicine treatment groups exhibited improved or alleviated cartilage degeneration, cell degeneration, synovial hyperplasia, and inflammatory cell infiltration in the ankle joint of MSU-induced mice.

## Discussion

Gouty arthritis, which is caused by delirious uric acid metabolism and MSU precipitation in one or more synovial joints, elicits local swelling, severe pain and dysfunction. MSU crystals are considered local proinflammatory stimuli that can initiate, strengthen and maintain an ardent inflammatory response [18]. The typical pathological characteristic of gout is that inflammatory cells, especially neutrophils, accumulate in both the synovial membrane and the joint fluid after being activated through signals released from surrounding macrophages [19]. MSU crystals interact with macrophages in the soft tissue of joints and stimulate the generation of protein complexes known as inflammasomes, which facilitate the conversion of pro-IL-1β into activated IL-1β. Afterwards, TNF-α is promoted by activated IL-1β and induces neutrophil influx into both the synovial tissue and the joint fluid [20]. Consequently, inhibiting the formation and activation of inflammasomes and proinflammatory cytokines, such as TNF-α and IL-1β, is an important therapeutic strategy to alleviate swelling, pain, and inflammation in inflammatory diseases, including gouty arthritis [17, 21].

The NLRP3 inflammasome, which forms through self-oligomerization between the CARD and PYD domains of NLRP3, pyrin and the adaptor ASC (apoptosis-associated speck-like protein containing a CARD), recognizes a cyclopedic range of damage-associated molecular patterns (DAMPs) as well as pathogen-associated molecular patterns (PAMPs) [22]. Upon activation by MSU, the NLRP3 inflammasome stimulates the transformation of procaspase-1 to activated caspase-1, which consequently cleaves the precursor cytokine pro-IL-1β into active proinflammatory IL-1β [23]. Some studies have confirmed that deficiencies in inflammasome components, such as NLRP3, ASC, and caspase-1, can block the activation of IL-1β in response to MSU stimulation [24, 25].

SHA is the total alkaloid prepared from the tuberous roots of *S. hainanensis*, and may include tetrahydroprotoberberine, aporphine, proaporphine, benzylisoquinoline and bisbenzylisoquinoline alkaloids [13, 26]. In our extracts of *S. hainanensis*, three alkaloids, including Xylopinine, Crebanine, and Plamatine, were identified by the LC-MS method. *S. hainanensis* is broadly used as a Chinese folk herb medicine for the treatment of inflammation, trauma, pain and fever, and SHA has also been reported to exert obvious anti-inflammatory effects in different inflammatory models, including cotton ball granuloma and egg white-induced paw swelling [27]. In this study, we detected the anti-inflammatory effects of SHA in a mouse model of MSU crystal-induced gouty arthritis by evaluating the ankle swelling and clinical scores in the paw tissue. The therapeutic effects were compared with those of colchicine, a popularly used anti-gout drug. We provide evidence for a gouty arthritis-related inflammation-relieving effect of SHA, which significantly reduced oedema and clinical scores in MSU crystal-induced mice. We also verified the anti-inflammatory effect of SHA on the production of proinflammatory cytokines (IL-1β and TNF-a), which are responsible for the MSU crystal-induced inflammatory gene expression.

Previous studies have proven the effects of NLRP3 and IL-1β in MSU crystal-induced gouty inflammation in a mouse model, and of IL-1α in recruiting neutrophils after intraperitoneal (IP) injection of MSU crystals [28, 29]. In our intraarticular injection model of MSU crystals, we did not detect significant differences in the serum levels of neutrophils and monocytes. This finding is in accordance with the results of Reber et al. in their WT and IL-1α−/− mice [30]. In contrast, local inflammatory cell infiltration was obvious in each group. Furthermore, our results indicated that the gene expression levels of *NLRP3*, *Caspase-1*, *IL-1β* and *TNF-α* were increased in MSU-induced mouse paw tissue and were reduced after SHA treatment. Correspondingly, the Western blotting results showed that the increased expression of NLRP3 was rescued by SHA. The downregulation of NLRP3 further inhibited the activation of caspase-1 and IL-1β. More importantly, we found that SHA effectively alleviated MSU-induced neutrophil infiltration in ankle joint tissues, with similar effectiveness to colchicine.

Collectively, our results indicate that SHA has inhibitory effects on inflammation and gouty arthritis primarily by intervening in the formation of inflammasomes and lessening the proinflammatory cytokines of TNF-α and IL-1β. As SHA is the total alkaloid obtained from *S. hainanensis*, further separation, purification and identification of active monomers are expected to provide new leading candidates for gouty arthritis therapy.

## Conclusion

Our in vivo model suggests for the first time that SHA alleviates MSU crystal-induced swelling in mcie and exerts anti-inflammatory effects by suppressing proinflammatory cytokine (TNF-α and IL-1β) release, NLRP3 inflammasome and caspase-1 activation, and inflammatory cell infiltration. These findings indicate the potential therapeutic effect of SHA that may suppress gouty arthritis through blocking the NLRP3/caspase-1 pathway and reducing swelling and inflammation.

## Supplementary Information

**Additional file 1: Supplementary Figure 1.** The original, unprocessed film pictures of NLRP3, Caspase-1, and IL-1 β expression in gouty ankle tissue of MSU- induced mice. Protein expression of NLRP3 and substrates in mouse ankle tissue was analysed by Western blotting after MSU crystals suspension (2.5 mg/50 ml) was injected intradermally for 48 h. The left side of the red dot line is the pictures provided in the main text. The right four strips are the normal liver, brain, heart, and kidney tissue of the normal mouse for control.

## Data Availability

Data are all contained within the paper.

## Abbreviations

SHA: *Stephania hainanensis* total alkaloid
MSU: Monosodium urate
IL-1β: Interleukin-1 beta
TNF-α: Tumor necrosis factor-alpha
NLRP3: NACHT LRR and PYD domain-containing protein 3
ASC: Apoptosis- associated speck-like protein containing a CARD
