# Supplementary figures and images for: Effects of Stephania hainanensis alkaloids on MSU-induced acute gouty arthritis in mice
Source: BMC Complement Med Ther. 2021 Jul 20;21:202. doi: 10.1186/s12906-021-03364-5 (PMC8293507; doi:10.1186/s12906-021-03364-5)

## Supplementary Figure 1

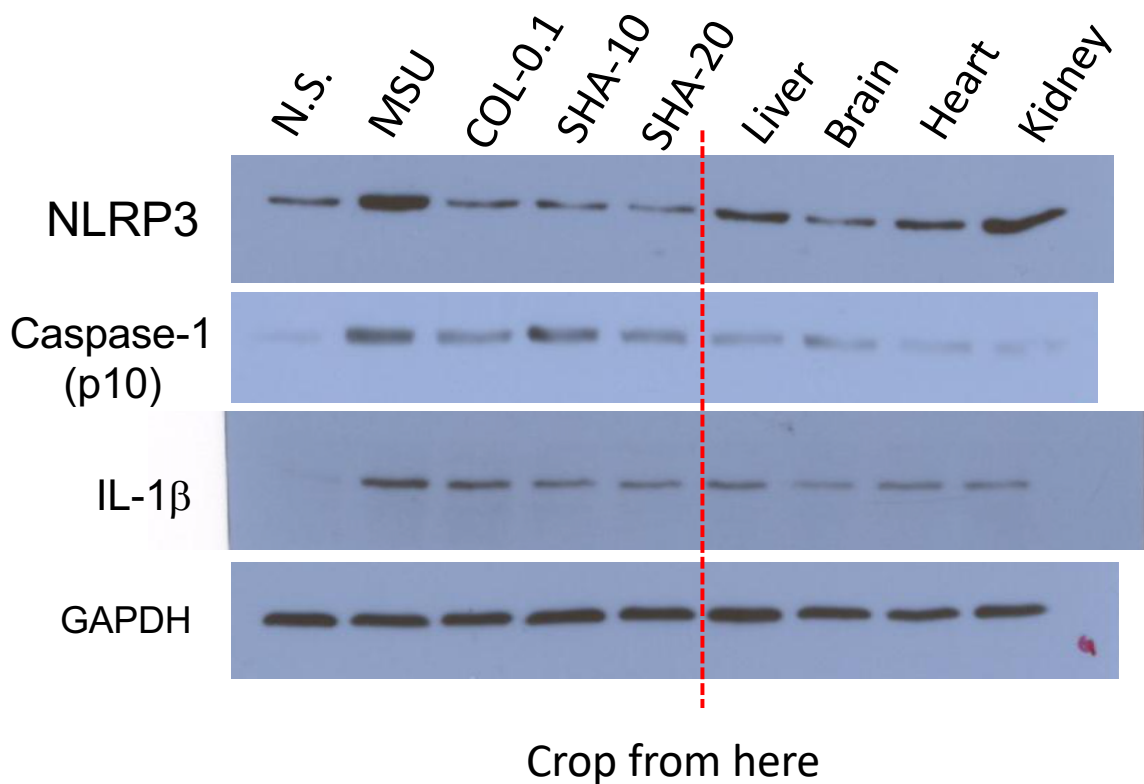

Supplement: Supplementary file 1 — Additional file 1: Supplementary Figure 1. The original, unprocessed film pictures of NLRP3, Caspase-1, and IL-1 β expression in gouty ankle tissue of MSU- induced mice. Protein expression of NLRP3 and substrates in mouse ankle tissue was analysed by Western blotting after MSU crystals suspension (2.5 mg/50 ml) was injected intradermally for 48 h. The left side of the red dot line is the pictures provided in the main text. The right four strips are the normal liver, brain, heart, and kidney tissue of the normal mouse for control. [file 12906_2021_3364_MOESM1_ESM.pdf]
